# Supplementary material for: Single-Cell Transcriptomic Analyses Define Distinct Peripheral B Cell Subsets and Discrete Development Pathways
Source: Front Immunol. 2021 Mar 18;12:602539. doi: 10.3389/fimmu.2021.602539 (PMC8012727; doi:10.3389/fimmu.2021.602539)
Supplement: Supplementary file 1 [file DataSheet_1.docx]

**Supplementary Figures**

Supplementary Figure S1


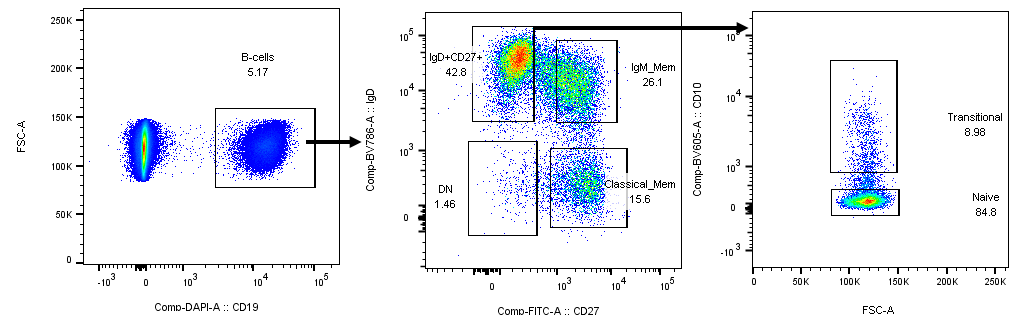


Gating strategies to sort B cell populations used in this work.

Supplementary Figure S2

Calculating composite transition score between cell clusters using RNA velocity streams overlaid on the dimensionality-reduced data space. (*left box*) As an illustration a single velocity stream (indicated by the arrow XY) was considered in a space with four clusters, the starting cluster (here coloured red, represented by the red dots [i.e. cells] around point X) and three other clusters represented by their centroids Z_1_, Z_2_ and Z_3_. (*middle*) For each of Z_1_, Z_2_ and Z_3_, we compute the angle θ between XY and the projection XZ_i_. A transition score (TS) was calculated based on this geometric relation, and based on this the transition which best aligns with XY was identified (in this case to the yellow cluster with centroid Z_2_). (*right*) Considering all arrows overlaid on the data space, a composite transition score (CTS) is calculated to quantify the support in terms of RNA velocity for transition between any pair of clusters, weighted by their distances.

Supplementary Figure S3

Expression of *CXCR5*, *FCRL4* and *ZEB2* across the four DN clusters.

Supplementary Figure S4


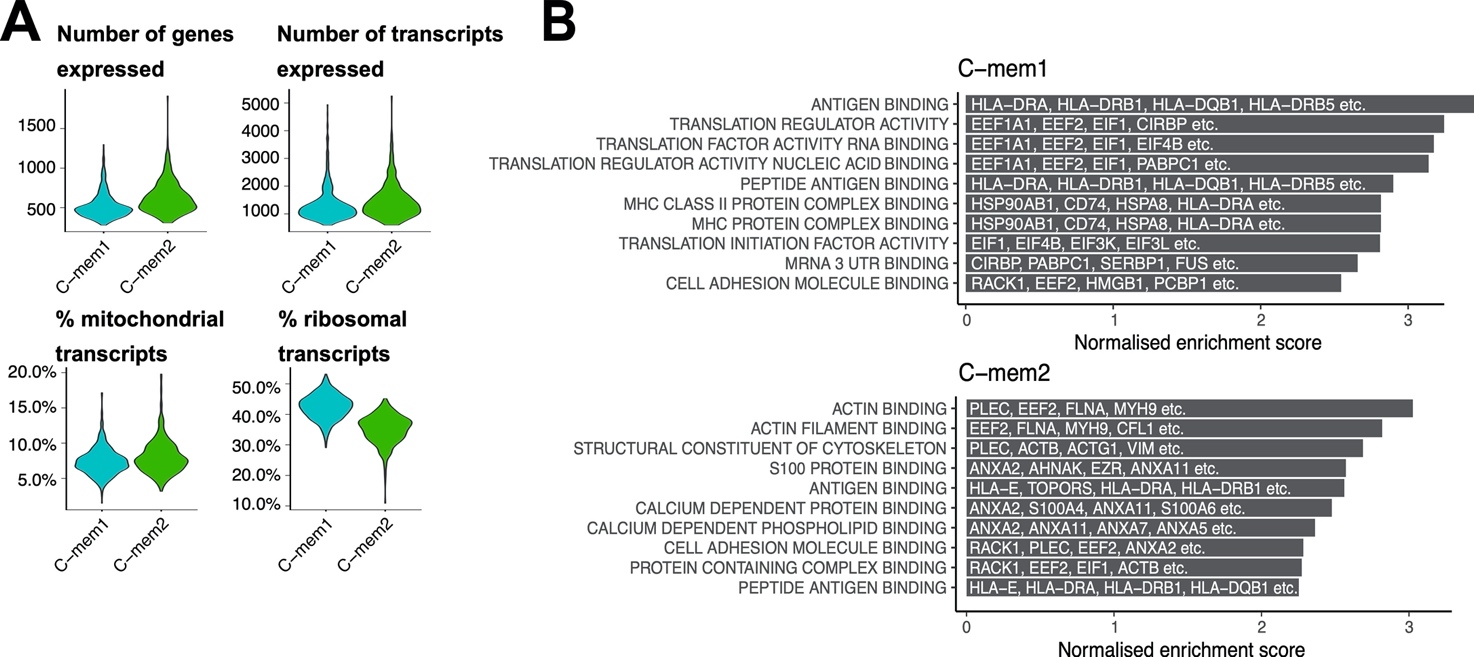


Comparison between C-mem1 and C-mem2 clusters. (A) Comparison of the number of genes and transcripts expressed, and the proportion of mitochondrial and ribosomal transcripts between the C-mem1 and C-mem2 clusters. (B) Gene set enrichment analysis of the inferred PPINs for the C-mem1 and C-mem2 cells. The top 10 significant pathways are shown here.

Supplementary Figure S5


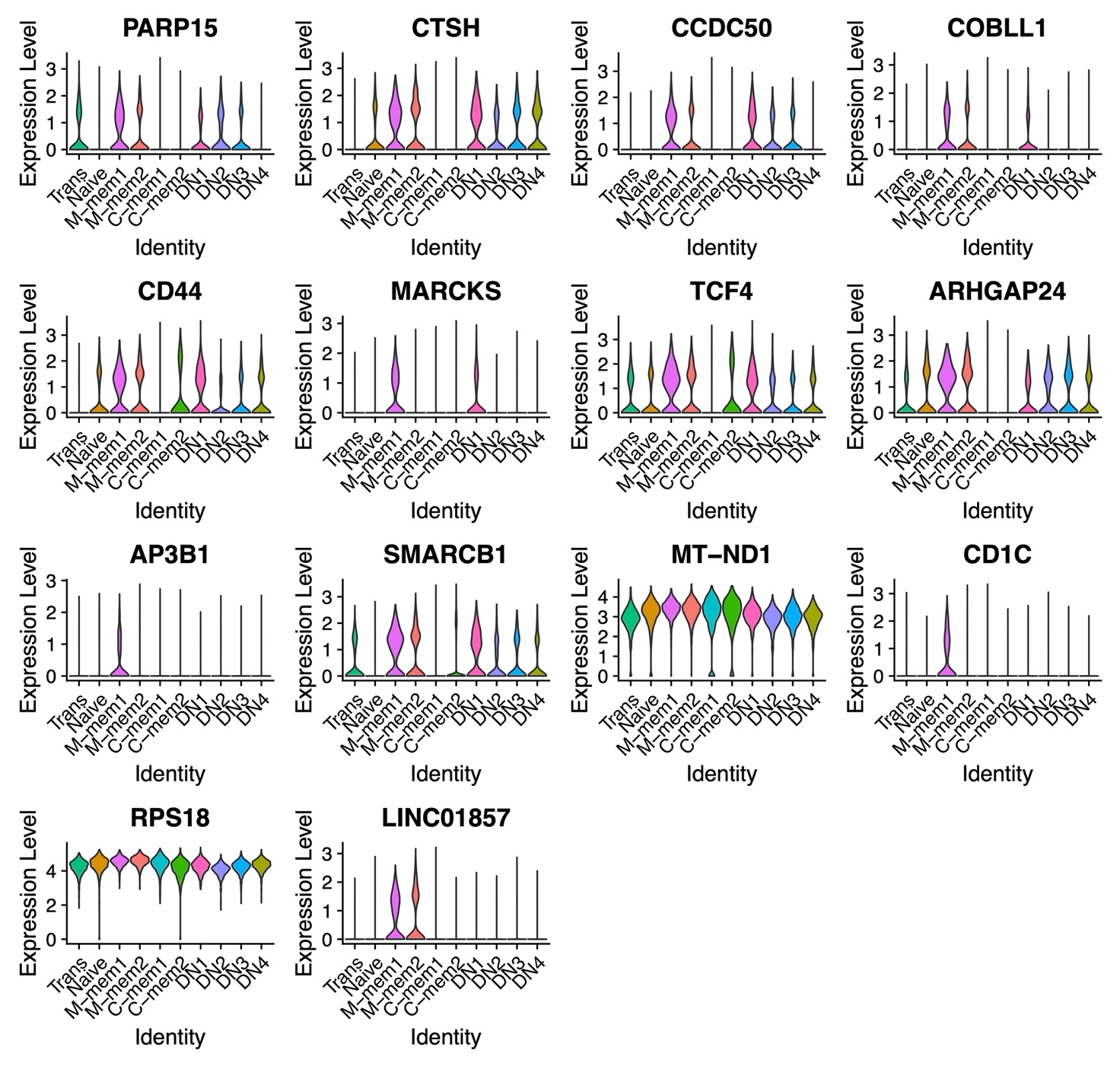


Expression of markers of IgM Memory cells shown in Figure 6A (main text), here depicted as violin plots across all cell clusters.

Supplementary Figure S6


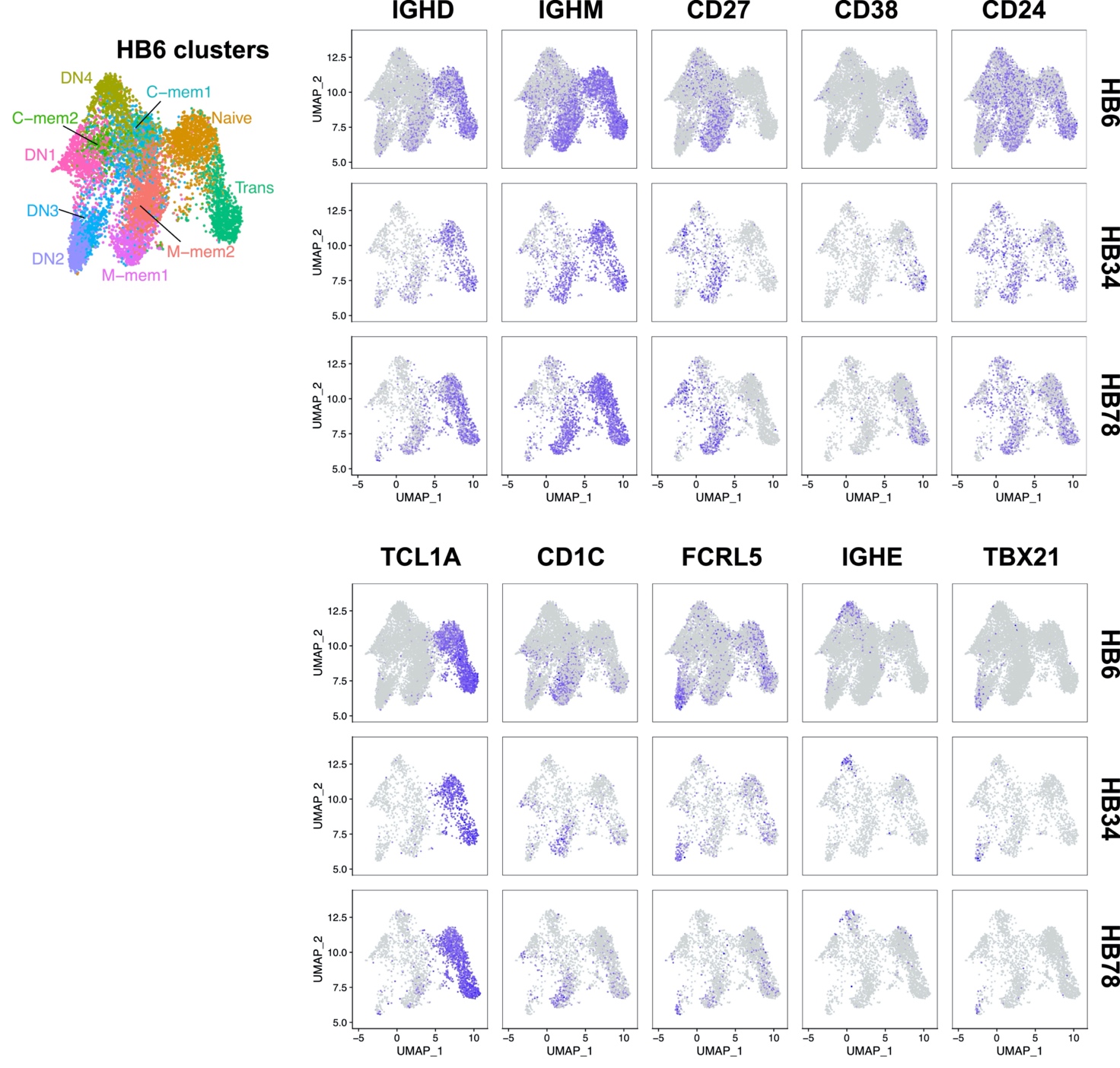


Expression of transcriptomic markers defined in this dataset. Cells from different individuals are shown in separate panels. Cluster definition derived from the HB6 samples is shown on the left (reproduced from Figure 7A) for reference.
